# Medical Statistics

**Published:** 1837-10

**Authors:** 


### MEDICAL STATISTICS.

### *Statistical Remarks on the Diseases peculiar to Women.* By S. TANCHOU.

M. TANCHOU entertained an opinion that the diseases peculiar to women were daily increasing; and, to convince himself of the fact, he examined the Bills of Mortality of Paris and the suburbs, which gave the following results:

In 1830, in the department of the Seine, 351 females died of sexual diseases; of which, 183 were cancer of the uterus.

| | | | | |
|---|---|---|---|---|
| In 1831, 379; | of which, | 246 | were cancer. |
| 1832, 396; | — | 230 | — |
| 1833, 498; | — | 250 | — |
| 1834, 436; | — | 304 | — |
| 1835, 508; | — | 285 | — |

Total, 2,568 females dead of sexual diseases; of which, 1,500 were cancer of the uterus.

M. T. enters into some discussions concerning the particular departments which furnish the greatest number of cases, and endeavours to assign the cause.

The following table, arranged according to the age, shows the period of life at which these diseases are most frequent:

Before twenty years, there were 25 cases of disease of the sexual organs, and not one of cancer.

| | | | | | |
|---|---|---|---|---|---|
| From 20 to 30, | 442 | sexual diseases, and | 86 | cancer |
| 30 .. 40, | 279 | — | 212 | — |
| 40 .. 50, | 137 | — | 402 | — |
| 50 .. 60, | 70 | — | 353 | — |
| 60 .. 70, | 60 | — | 242 | — |
| 70 .. 80, | 42 | — | 147 | — |
| 80 .. 90, | 13 | — | 58 | — |

By this we see that, during the first twenty years of life, cancer of the sexual organs is unknown, and that even other diseases of these parts are rare; whilst, from twenty to thirty, cancer increases; and that, from thirty to forty, the cases of cancer have increased so much as to be nearly equal to the cases of sexual disease.

*Journal des Connaissances Médicales. November,* 1836.

